# Pleiotropic effects of melanin pigmentation: haemoparasite infection intensity but not telomere length is associated with plumage morph in black sparrowhawks

**DOI:** 10.1098/rsos.230370

**Published:** 2024-04-03

**Authors:** Edmund Rodseth, Petra Sumasgutner, Gareth Tate, Johan F. Nilsson, Hannah Watson, Michelle F. Maritz, Robert A. Ingle, Arjun Amar

**Affiliations:** ^1^ Department of Molecular and Cell Biology, University of Cape Town, Cape Town, South Africa; ^2^ FitzPatrick Institute of African Ornithology, University of Cape Town, Cape Town, South Africa; ^3^ Konrad Lorenz Research Centre, Department of Behavioural and Cognitive Biology, University of Vienna, Vienna, Austria; ^4^ Birds of Prey Programme, Endangered Wildlife Trust, Midrand, South Africa; ^5^ Evolutionary Ecology, Department of Biology, Lund University, Lund, Sweden

**Keywords:** polymorphism, pleiotropy, haemoparasite, *Accipiter*, melanin, telomere

## Abstract

There is increasing recognition of the potential pleiotropic effects of melanin pigmentation, particularly on immunity, with reports of variation in haemoparasite infection intensity and immune responses between the morphs of colour-polymorphic bird species. In a population of the black sparrowhawk (*Accipiter melanoleucus*) in western South Africa, light morphs have a higher haemoparasite infection intensity, but no physiological effects of this are apparent. Here, we investigate the possible effects of haemoparasite infection on telomere length in this species and explore whether relative telomere length is associated with either plumage morph or sex. Using quantitative polymerase chain reaction analysis, we confirmed that dark morphs had a lower haemoparasite infection intensity than light morphs. However, we found no differences in telomere length associated with either the haemoparasite infection status or morph in adults, although males have longer telomeres than females. While differences in haemoparasite intensity between morphs are consistent with pleiotropic effects of melanin pigmentation in the black sparrowhawk, we found no evidence that telomere length was associated with haemoparasite infection. Further work is needed to investigate the implications of possible pleiotropic effects of plumage morph and their potential role in the maintenance of colour polymorphism in this species.

## 1. Introduction

Pleiotropy is a phenomenon in which a single gene affects more than one physiological process [1]. It is of interest in the study of phenotypic polymorphism, as the underlying genetic polymorphism associated with a particular trait may have other effects on seemingly unrelated physiological processes [2,3]. Pigmentation, particularly melanin-based pigmentation, may be a pleiotropic trait in many species, as differences in melanin pigmentation are associated with variation in several physiological and behavioural traits, including sexual behaviour, aggression, energy homeostasis and immune function in a variety of vertebrates, including birds [3–5]. It is thus important to investigate possible pleiotropic effects that the underlying genetic pathways of melanogenesis may have on other physiological processes besides plumage coloration to better understand the trait’s potential adaptive function and explain its maintenance in a population [2,3].

A proposed mechanism for the covariance between melanin pigmentation and other traits, particularly immunity, involves pleiotropic effects of the melanocortin system [3]. The melanocortin G-protein-coupled receptors bind to α-, β- and γ-melanocyte-stimulating hormones (MSHs) and adrenocorticotropin hormone (ACTH). The binding of MSHs and ACTH to various melanocortin receptors affects several physiological processes besides pigmentation, such as inflammation, hypothalamic–pituitary–adrenal (HPA) axis stress response and the autoimmune response [3]. The pleiotropic effects on immune function have elicited considerable research interest [2]. For example, barn owl (*Tyto alba*) nestlings with darker-coloured parents were able to mount a stronger immune response to an immune challenge than nestlings with lighter-coloured parents [6]. In feral pigeons, darker individuals were found to have lower haemosporidian (blood parasites including *Haemoproteus, Plasmodium* and *Leucocytozoon* species) infection intensities and higher phytohaemagglutinin (PHA)-induced inflammatory responses than lighter individuals [7]. Conversely, in the Eleonora’s falcon (*Falco eleonorae*), melanism was associated with a reduced PHA-induced inflammatory response in male nestlings [8], and dark morph birds had a higher prevalence of *Plasmodium* parasite infection than pale morphs [9]. In the tawny owl (*Strix aluco*), grey morphs exhibited a stronger immune response to haemosporidian parasites and had lower infection intensities than rufous morphs [10].

Melanin pigmentation may also affect telomere dynamics, which may be related to the link between melanin pigmentation and immunity. Telomeres are short tandem repeat sequences found in nucleoprotein complexes at chromosomal ends [11,12] that function as protective caps to maintain chromosomal DNA integrity, particularly during the replication process [13–15]. Additionally, they are believed to prevent broken chromosomes from rearranging and fusing and are involved in chromosome segregation [16–18]. Telomeres shorten with each round of DNA replication and cell division, due to the inability of DNA polymerase to replicate to the 3′-end of the lagging strand during DNA replication [19]. When the telomere length shortens to a critical threshold, the cell begins to senesce, a process which eventually ends in apoptosis [11].

Telomere shortening is strongly linked to senescence and is often used as a proxy for age or ageing [20–23]. However, the rate of telomere shortening can differ between individuals of the same species, and other factors may also be involved in telomere shortening, such as exposure to adverse environmental conditions or various other sources of physiological stress (including oxidative stress), not just age [24–29]. For example, nestling great tits (*Parus major*) raised in an urban environment had significantly shorter telomeres than those raised in a rural environment, independent of their genetic origin [30]. Telomere length in early life can also have carry-over effects on survival; for example, in the European storm petrel (*Hydrobates pelagicus*), nestlings reared under unfavourable conditions had shorter telomere lengths later in life, and nestlings with shorter telomeres early in life had higher mortality rates [31]. Thus, shorter telomeres or a faster rate of telomere shortening are indicative of physiological stresses in many vertebrates. However, it is not yet clear whether a short telomere length imposes a direct effect on individual fitness, or instead, it is an indirect biomarker of physiological conditions or various physiological stresses experienced by the individual, such as severe infections, the presence of predators or adverse conditions or activities that require physical exertion, such as breeding [22,32,33].

Telomere length variation in birds can also be associated with infections caused by haemosporidian blood parasites [32,34]. Such infections are common in birds, and while their physiological effects in wild bird populations are unclear, some experimental studies have shown a negative correlation between infection intensity and host reproductive success [35,36] or survival [37,38]. Uninfected male blue tits (*Cyanistes caeruleus*), as well as individuals infected with a less severe disease-causing *Haemoproteus* parasite, were found to have longer telomeres than individuals infected with the more severe *Plasmodium* parasite [34]. Even chronic, low-level haemoparasite infection can be associated with greater telomere attrition, reduced lifespan, lower reproductive output and reduced quality of offspring, as seen in the great reed warbler (*Acrocephalus arundina*) [32].

Furthermore, melanin pigmentation is linked to telomere dynamics in several bird species [39,40]. For example, adult rufous morph tawny owls with more pheomelanin in their plumage have shorter telomeres than grey morphs and experience faster telomere shortening [39,41]. In the same species, haemoparasite infection status was also associated with shorter telomeres [39], and morph-specific differences in lifespan were also detected, with grey morphs having higher survival rates and higher lifetime reproductive output under harsh winter conditions [42,43]. Thus, there may be a link between melanin pigmentation and telomere dynamics mediated through the pleiotropic effects of genes involved in the melanin production pathway [3]. Melanin biosynthesis can cause an increase in reactive oxygen species [41,44], which may cause telomere shortening through increased oxidative stress [45,46]. Indeed, it has been found that an increase in antioxidants in the diet can reduce pathogen-induced telomere attrition in certain species [47]. However, note that although oxidative stress is known to cause telomere shortening *in vitro* in cell cultures, it is not yet known whether it is associated with telomere shortening *in vivo* at physiological oxidative stress levels [48,49].

In this study, we investigate the potential pleiotropic effects of melanin pigmentation on immunity and telomere length in the colour-polymorphic black sparrowhawk (*Accipiter melanoleucus*). The species is long-lived, with individuals more than 10 years old recorded in the study population, an estimated average lifespan of 4 to 5 years and an estimated annual adult survival rate of 89% [50,51]. Throughout the range of this species, adult birds occur in two plumage morphs, light and dark, which are unrelated to sex and differ in the extent of white plumage on the chin, throat, breast and flanks. Light and dark morph individuals do not consistently differ in size and do not show differences in survival or reproductive productivity [52]. Previous studies found no difference in the prevalence of haemoparasite infection between morphs, but showed that dark morph birds in the Western Cape province of South Africa had a lower infection intensity of the haemosporidian parasite *Haemoproteus nisi* than light morphs [53,54]. It has been proposed that the increased frequency of dark morphs in this region may result from positive selection on their enhanced ability to control haemoparasite infection, though McCarren *et al*. [54] found no obvious fitness impacts of haemoparasite infection in terms of productivity or survival. However, there may still be less obvious impacts of infection, and as telomere attrition has been associated with chronic haemoparasite infection in other species, it may serve as a biomarker for the negative physiological effects of such infections. Here, we used a quantitative polymerase chain reaction (PCR)-based approach to examine the effect of haemoparasite infection prevalence and intensity on relative telomere length. We also explore whether this species exhibits sex-specific or morph-specific telomere dynamics that might indicate pleiotropic effects of the genes involved in determining plumage morph.

## 2. Material and methods

### 2.1. Study population and DNA extraction

Blood samples were collected from 89 adult black sparrowhawks (44 males and 45 females; 45 dark morphs and 44 light morphs) between 2009 and 2019 in an area across South Africa between 25.52° S to 34.70° S and 19.70° E to 33.00° E. Twelve of these individuals had also had blood collected as nestlings and were thus of known age when resampled, while the other 77 birds were of unknown age when sampled. Adult territories were located by surveying suitable stands of trees during the breeding season (March to November) for black sparrowhawk vocalization, whitewash or prey remains. Wild adults were trapped on their territories using bal-chatri traps baited with live white feral pigeons (*Columba livia*), while chicks were sampled directly from the nest. Blood samples were drawn from the medial cubital vein or the medial metatarsal vein, and were either stored under 100% (v/v) ethanol at 4°C or in 0.5 M EDTA at −18°C. DNA was extracted from the whole blood samples using a modified salt/chloroform DNA extraction protocol described by Kanai *et al.* [55], and DNA integrity was assessed by agarose gel electrophoresis.

### 2.2. Quantitative polymerase chain reaction determination of relative haemoparasite infection intensity and telomere length

For the quantification of apicomplexan haemoparasite infection intensity, an 85 bp fragment of the plastid-like large subunit ribosomal-RNA (*LSU-rRNA*) was amplified using the primers Plasmo474for (5′-AGGCTAATCTTTTCCGAGAGTCC-3′) and Plasmo558rev (5′-ACATACTACTGCTTTAGGATGCGA-3′) [56]. These primers do not distinguish between *Haemoproteus, Plasmodium* and *Leucocytozoon* species, so the infection intensities calculated potentially represent the intensity of infection by parasites from all three genera, although *Plasmodium* infection has never previously been reported in this population [53,54,57]. A standard curve for these assays was generated from a 595 bp fragment of the *Plasmodium falciparum* genome (containing the 85 bp region) amplified from the *P. falciparum* genomic DNA using the primers L1 (5′-GACCTGCATGAAAGATG-3′) and L2 (5′-GTATCGCTTTAATAGGCG-3′) [58].

Relative telomere length (RTL) was measured by quantitative polymerase chain reaction (qPCR using the primers Tel1b (5′-CGGTTTGTTTGGGTTTGGGTTTGGGTTTGGGTTTGGGTT-3′) and Tel2b (5′-GGCTTGCCTTACCCTTACCCTTACCCTTACCCTTACCCT-3′) [59]. Finally, a 114 bp genomic region which contains a single copy nuclear non-coding sequence that is highly conserved across vertebrates [60] was amplified using the primers sfsr/3Fb (5′-ACTAGCCCTTTCAGCGTCATGT-3′) and sfsr/3Rb (5′-CATGCTCGGGAACCAAAGG-3′) [61]. This region has been used to normalize qPCR data in numerous studies involving parasite infection intensity and telomere length quantification in birds [32,39,61–63].

The KAPA SYBR^®^ FAST qPCR Kit (Kapa Biosystems) was used for all qPCR analyses, as per the manufacturer’s instructions. Two nanograms of DNA extracted from whole blood samples was added to each reaction for haemoparasite quantification and 0.1 ng of DNA was used for RTL quantification. qPCR was performed using a Corbett Rotor-Gene 6000 HRM Real-Time PCR machine (Qiagen) with the following parameters: 95°C for 3 min, 35 × (95°C for 3 s, 60°C for 20 s and 72°C for 1 s) and 72°C for 90 s. This was followed by ramping the temperature from 72 to 90°C to generate a melt curve. A standard curve with eight points spanning a dilution range of 1 to 1/128 (sfsr), seven points spanning a dilution range of 1 to 1/64 (RTL), or eight points spanning a dilution range of 1 to 1/2187 (haemoparasite infection intensity) was included in every qPCR run. Only qPCR runs showing a single product in the melt curve and where the efficiency of the standard curve was between 0.9 and 1.1 with an *R* ^2^ value greater than 0.99 were considered successful (electronic supplementary material, table S1). Two or three technical replicates per biological sample were included and samples where the technical replicates showed a difference in *C*_q_ value greater than 0.5 were rerun. The calculated concentration values for the technical replicates were used to generate coefficient of variation (CV) values for each biological sample. These values are provided in the experimental data file [64], while the average CV for the biological samples in each qPCR run is provided in the electronic supplementary material, table S1. The relative haemoparasite infection intensity and RTL values were calculated from the standard curves and normalized to the single-copy nuclear non-coding region (sfsr/3Fb sfsr/3RB amplicon) in the same DNA sample. Parasite prevalence was scored either 0 or 1 depending on whether amplification of the parasite 85 bp fragment was detected or not. In addition, a blood slide was prepared from 68 out of the 89 blood samples for haemoparasite quantification by microscopy using the method described by McCarren *et al.* [54] to determine the number of cells infected by *Haemoproteus* or *Leucocytozoon* per 10 000 erythrocytes as a measure of infection intensity to validate our qPCR results.

### 2.3. Statistical analyses

To assess body condition while accounting for differences in body size, a condition index (CI) was derived by extracting the residuals of a linear regression with body mass as the response variable and tarsus length and sex as fixed variables for the 69 individuals for which these measurements were available. This provides an effective method for quantifying condition while accounting for differences in body size [65–67].

To confirm that our qPCR analysis of haemoparasite infection in black sparrowhawks was reliable, we compared the intensity values obtained using qPCR with those obtained by microscopy on blood slides for 68 samples. This was done using a linear regression model with haemoparasite infection intensity values as determined by microscopy (the number of cells infected by *Haemoproteus* and *Leucocytozoon* per 10 000 erythrocytes) as the explanatory variable and qPCR haemoparasite infection intensity values as the response variable.

For all of the following analyses, only parasite prevalence and intensity values generated by qPCR were used. We explored whether morph or sex was significantly associated with (i) haemoparasite prevalence, (ii) haemoparasite intensity or (iii) RTL. The samples were collected over several years over a large geographical area, and, as such, we recognized that other variables may also be associated with haemoparasite prevalence and intensity and RTL, and so took these variables into account in the analysis. These variables consist of the longitude at which the bird was captured (as the rainfall seasonality across the species’ range differs largely with longitude), whether the bird was captured during the peak breeding season (May to September, inclusive [67]), year of capture and qPCR run ID. Of these, all variables were treated as fixed effects except for the year of capture and qPCR run ID, which were included as random effects in all models except for the models that failed to converge, in which case they were treated as fixed variables. In the descriptions of the models used below, these variables will be referred to as ‘longitude’, ‘breeding’, ‘year’ and ‘qPCR run’, respectively. We also explored whether the body condition (CI) was associated with haemoparasite prevalence or infection intensity or with RTL in a subset of the samples for which the CI was available.

For several of the following analyses, to explore the model space and identify the most parsimonious model, we performed model selection using the dredge function from the MuMIn package [68]. The dredge function exhaustively explores multiple model combinations based on Akaike’s information criterion corrected for small sample sizes (AICc). Model selection aimed to identify the most informative predictors contributing to the variation in the response variable [69,70]. See the electronic supplementary material for the full details of models used, model selection tables and AICc values.

For models including CI as a variable, only individuals for which the CI was available were included in these analyses (69 individuals). For models including infection intensity and CI as variables, only individuals with an infection intensity greater than 0 (i.e. individuals for which the parasite 85 bp fragment were detected using qPCR) and for which the CI was available were included in these analyses (50 individuals). For models including infection intensity (but not CI) as a variable, only individuals with parasite infection intensity values greater than 0 were included (64 individuals).

Additionally, to test whether any effects were driven by outliers in the RTL or haemoparasite infection intensity values, models with these variables were rerun with outliers removed. Samples were considered outliers if the natural logarithm of the haemoparasite infection intensity (measured by qPCR) or RTL was greater than the third quartile or less than the first quartile by more than 1.5 times the interquartile range. Using these criteria, two outliers were removed based on high infection intensities (log haemoparasite infection intensity greater than 8), and one outlier was removed based on a high RTL value (RTL > 3).

#### 2.3.1. Haemoparasite prevalence

Initially, we attempted to use a generalized linear mixed-effects model with the binary response of infected (1) or not infected (0) fitted with a binomial error structure and logit link function, with morph, sex, longitude and breeding as predictor variables, and year and qPCR run as random variables. An interaction between morph and sex was included in the model. However, the model failed to converge. We then fitted a generalized linear model (GLM) with the same parameters except with year and qPCR run as predictor variables. We performed model selection based on AICc values, and the null model (intercept only) was identified as the top-ranked model. Finally, we repeated the analysis on a subset of the data for which the CI was available to check whether including this variable had any material effect on the results. The null model (intercept only) was again identified as the top-ranked model.

#### 2.3.2. Haemoparasite infection intensity

For haemoparasite infection intensity, only infected individuals (haemoparasite infection intensity measured by qPCR greater than 0) were included in the analysis. We used a linear mixed model (LMM) with log-transformed infection intensity as the response variable, with morph, sex, longitude and breeding as predictor variables, and year and qPCR run as random variables. An interaction between morph and sex was included in the model. We performed model selection based on AICc values, and the best-fit model was identified as a linear model (LM) with log-transformed infection intensity as the response variable and with morph as the sole predictor variable. We then reran this best-fit model on a subset of the data with outliers removed. Lastly, we reran this best-fit model on the subset of the data for which the CI was available, to check whether including this variable had any material effect on the results.

#### 2.3.3. Relative telomere length

For the 12 birds where both an adult and a nestling blood sample were available, we explored how telomere length varied between the nestling and adult life stages, and whether telomere attrition differed between the sexes or with age. To explore how telomere length differed between adults and nestlings, we fitted a LMM with the bird ID as a random term and RTL as the response variable, with age (adult or nestling) as the predictor variable.

To explore the factors that may influence telomere attrition in these 12 samples, we fitted a linear model (LM) with telomere attrition (nestling RTL − adult RTL) as the response variable and age (in years at the time the adult sample was taken), adult haemoparasite infection status and adult haemoparasite infection intensity as predictor variables. To account for the regression to the mean effect, we also included the baseline telomere length (measured at the nestling stage) as a predictor variable [71]. We performed model selection based on AICc values, and the best-fit model was identified as a LM with baseline telomere length as the sole predictor variable.

To explore the variables associated with RTL in the 89 adult samples, we used a LMM with RTL as the response variable, haemoparasite infection status (infected or uninfected), morph, sex, longitude and breeding as predictor variables, and the year and qPCR run as random effects. The model included an interaction between morph and sex. We performed model selection based on AICc values, and the best-fit model was identified as a linear model with RTL as the response variable and sex as the sole predictor variable. To explore the possible association between haemoparasite infection intensity and RTL, we reran this best-fit model with infection intensity included as a predictor variable. We then reran this best-fit model (linear model with RTL as the response variable and sex as the sole predictor variable) on a subset of the data with outliers removed. Lastly, we reran this again on the subset of the data for which the CI was available to check whether including this variable had any material effect on the results.

All models were implemented in R v. 3.5.3 [72]. LMMs were implemented using the lme4 package [73]. The models were evaluated based on AICc, which was calculated using the MuMIn package [68]. The details on the models used can be found in electronic supplementary material, S2. Interactions are only reported where they are significant.

## 3. Results

### 3.1. Haemoparasite prevalence and intensity

**Figure 1.**
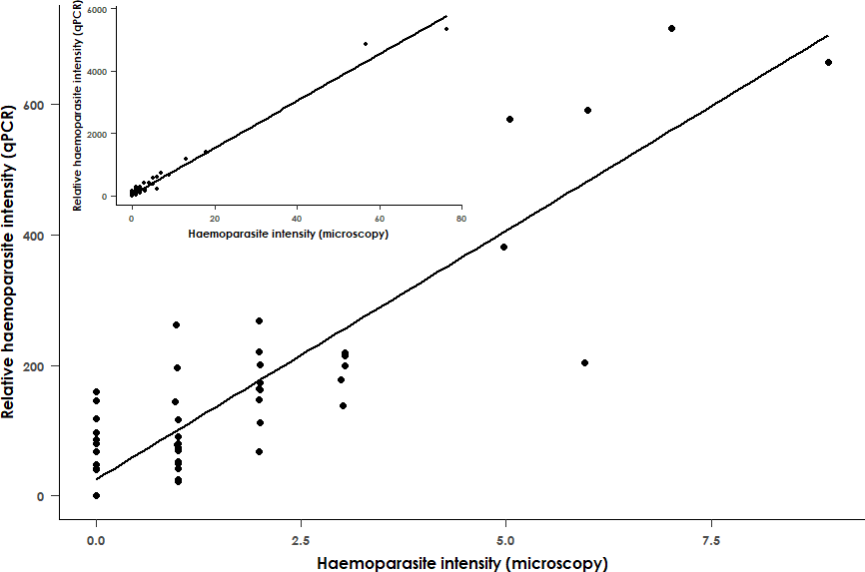
Relationship between haemoparasite infection intensity as determined by microscopy and qPCR in adult black sparrowhawks. Haemoparasite infection intensity determined by microscopy (number of cells infected by *Haemoproteus* or *Leucocytozoon* per 10 000 erythrocytes) is significantly positively correlated with that determined by qPCR (*R* ^2^ = 0.98, *F*_1,66_ = 3826 and *p* < 0.001). The main figure excludes all samples for which the haemoparasite intensity estimated by microscopy is greater than 10 to better visualize the relationship. (Inset: full figure, including all samples. *n* = 68).

There was a significant positive correlation between our qPCR and microscopy measurements of the intensity of haemoparasite infection (*R* ^2^ = 0.98, *F*_1,66_ = 3826 and *p* < 0.001, figure 1), confirming that our use of qPCR to determine the prevalence and intensity of infection in this system mirrored the results obtained by microscopy. Apicomplexan haemoparasites (*Haemoproteus* and/or *Leucocytozoon*) were detected in 64 out of 89 birds (a 72% prevalence rate).

**Figure 2.**
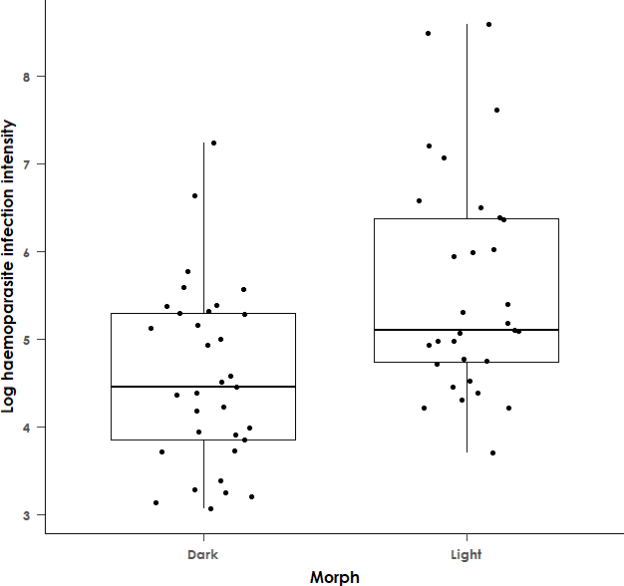
Box plot showing the natural logarithm of haemoparasite infection intensity (determined by qPCR) in dark and light morph adult black sparrowhawks. Light morph birds have significantly higher haemoparasite infection intensity than dark morph individuals (*F*_1,62_ = 12.48 and *p* < 0.001). Boxes show the median and interquartile range, while the upper and lower whiskers extend to the highest or lowest value within 1.5 times the interquartile range. *n* = 64.

Examining the relationship between haemoparasite infection prevalence and the hypothesized predictor variables during the model selection process, the null model (intercept only; AICc = 107.7) was identified as the best-fit model. This suggests that none of the predictor variables, including plumage morph or sex, are significantly associated with haemoparasite infection status in this species. When the analysis was repeated with the condition index included in the model, the null model was still identified as the best-fit model (AICc = 83.3), and thus the addition of the CI did not materially change the results. In contrast, infection intensity was significantly associated with morph (*F*_1,62_ = 12.48 and *p* < 0.001, figure 2), with a model including morph as the sole predictor variable identified as the best-fit model using AICc values, which suggests that the other variables, including sex and the CI, are not associated with infection intensity. Higher infection intensities were observed in light morphs compared with dark morphs. The association between morph and infection intensity was still significant when models were rerun excluding outliers for infection intensity (*F*_1,60_ = 9.66 and *p* = 0.003). Finally, the CI was not significantly associated with infection intensity (*F*_1,47_ = 3.68 and *p* = 0.06).

### 3.2. Relative telomere length analysis

**Figure 3.**
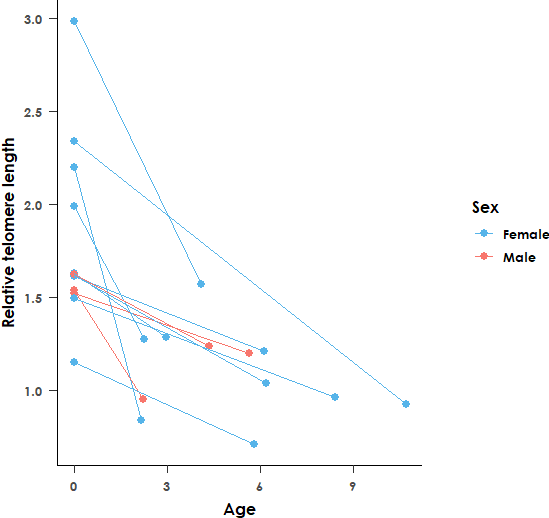
Relative telomere length (determined by qPCR) of black sparrowhawks as nestlings versus adults. The age at which individuals were resampled is shown on the *x*-axis. Lines connect the same individuals sampled at two different points in time. Adults have significantly shorter telomeres than nestlings (*χ* ^2^_1, 24_ = 32.47 and *p* < 0.001). Sex of the individuals is indicated by colours (blue = female and red = male). *n* = 12.

Exploring RTL in the 12 paired samples (nine females and three males) with nestling and adult blood samples showed that adults had significantly shorter telomeres than nestlings (*χ* ^2^_1,24_ = 32.47 and *p* < 0.001, figure 3). Despite the difference in RTL between nestlings and adults, when considering telomere attrition (telomere length as a chick – telomere length as an adult), the age of the adult when resampled was not included in the best-fit model for telomere attrition, and is thus unlikely to be associated with the degree of telomere shortening. The best-fit model for telomere attrition (AICc = 4.1) included the baseline telomere length as the sole predictor variable, suggesting that age and haemoparasite infection prevalence and intensity are not associated with telomere attrition in the species.

**Figure 4.**
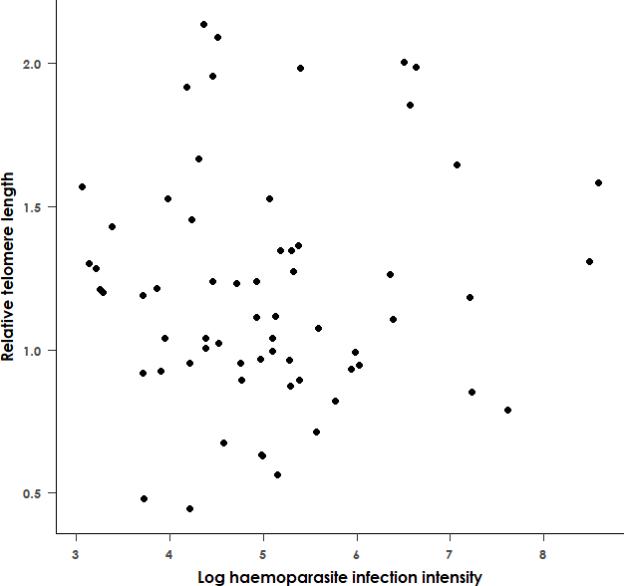
Relationship between the relative telomere length (determined by qPCR) and the natural logarithm of the haemoparasite infection intensity (determined by qPCR) in infected adult black sparrowhawks. The haemoparasite infection intensity was not significantly associated with relative telomere length (*F*_1,62_ = 0.02, *p* = 0.89). *n* = 64.

**Figure 5.**
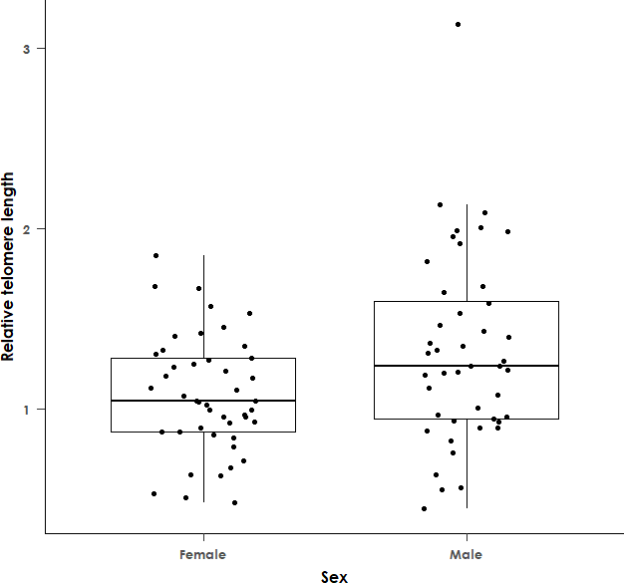
Relative telomere length (determined by qPCR) in adult female black sparrowhawks versus males. The difference between the sexes was significant, with males having longer telomeres than females (*F*_1,87_ = 6.73 and *p* = 0.011). The boxes show the median and interquartile range, while the upper and lower whiskers extend to the highest or lowest value within 1.5 times the interquartile range. *n* = 89.

**Figure 6.**
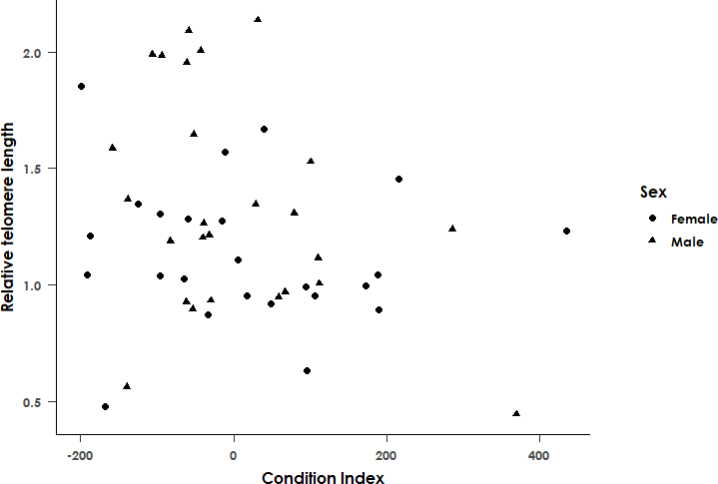
Relationship between the relative telomere length (determined by qPCR) and the condition index in adult black sparrowhawks. Sex of the individuals is indicated by point shapes. Relative telomere length is significantly negatively associated with the condition index (*F*_1,64_ = 5.32 and *p* = 0.024). *n* = 69.

Within our sample of 89 adult black sparrowhawks, the best-fit model for RTL (AICc = 97.6) included sex as the sole predictor variable, suggesting that RTL is not associated with haemoparasite prevalence, morph or any of the other hypothesized predictor variables. Similarly, in infected individuals, RTL is not associated with infection intensity (*F*_1,61_ = 0.02 and *p* = 0.89, figure 4). We did, however, find differences in RTL between sexes, with males having significantly longer telomeres than females (*F*_1,87_ = 6.73 and *p* = 0.011, figure 5). This association was still significant when the model was rerun excluding outliers in RTL (*F*_1,84_ = 4.94 and *p* = 0.029). Additionally, condition index was found to be significantly negatively associated with telomere length in the subset of data for which these measurements were available (*F*_1,66_ = 5.32, *p* = 0.024 and adjusted *R* ^2^ = 0.081, figure 6), with individuals with a lower mass than expected given tarsus length and sex having a higher RTL. This association was still significant when the model was rerun excluding outliers in RTL (*F*_1,64_ = 4.65 and *p* = 0.038).

## 4. Discussion

Using a qPCR-based approach as an alternative to more traditional blood slide analyses, our results confirmed those of Lei *et al*. [53], which showed haemoparasite prevalence to be similar between morphs and sexes in black sparrowhawks, but that light morphs had a higher haemoparasite infection intensity. Our results therefore further reinforce the idea that the genes involved in determining adult plumage morph in this species may be pleiotropic with respect to immunity, specifically the ability to control haemoparasite infection intensity.

It is possible that dark morph birds can control haemoparasite infection more effectively than light morph birds. Alternatively, light morph birds may cope with infection intensities that would kill dark morph birds, resulting in the selective removal of dark morph birds with high infection intensities from the population. However, given the fact that haemoparasite prevalence in this population is high and unrelated to morph (both morphs are equally likely to be infected), and the fact that survival in this population is unaffected by plumage morph [52], it seems unlikely that dark morph individuals with high infection intensities are selectively removed from the population. Moreover, it is unclear whether haemoparasite infections are often fatal—in a recent study on haemoparasite infection in a related species, the European sparrowhawk (*Accipiter nisus*), no clear cases of haemoparasite-induced mortality were seen [74].

To investigate the possible physiological effects of haemoparasite infection intensity, we used the relative telomere length (RTL) as a biomarker for the physiological cost of infection. A major limitation of our study is that the effects that ageing may have on telomere dynamics in the black sparrowhawk are not yet understood. The majority of individuals we sampled were of unknown age, and thus it was not possible to control for the possible effects of age on telomere length. As such, our analyses involving telomere length are predicated on the assumption that differences in RTL between individuals are not due to age. Our analysis of the small subset of individuals whose ages were known (12 birds) provides some support for this assumption. In this subset, while nestlings had significantly longer telomeres than adults, the age of adults at resampling was not associated with telomere attrition in birds of up to 10 years of age, which encompasses the average lifespan of 4–5 years for this species [51]. This suggests that black sparrowhawks could go through a period of telomere attrition during the transition from nestling to adulthood, but that further telomere attrition happens very slowly, if at all, in adults. Similar results have been reported in the European shag (*Phalacrocorax aristotelis*), wandering albatross (*Diomedea exulans*) and jackdaw (*Corvus monedula*), and the pattern of telomere shortening where telomere lengths shorten in the transition to adulthood but then remain similar over the course of their adult lifespan may be widespread in birds [75–78]. This does suggest that the telomere length in adult black sparrowhawks could be used as a useful proxy for telomere attrition due to stressors, such as haemoparasite infection, and that the age of the individuals, which was unknown for 77 out of the 89 individuals sampled, may not be a significant confounding factor in our analyses. Nevertheless, due to the small number of individuals of known age available, this result does not eliminate the possibility that age could be a confounding factor in our analyses, which would require further work focused on telomere dynamics and age in this species to verify. As such, our results must be regarded as tentative until such studies are conducted. Regarding the possibility that dark and light morph birds sampled could have different average ages (which could affect analyses involving morphs), previous studies have shown no morph-specific effects on survival in this population [52], suggesting that there is no reason to suspect that the dark and light birds sampled had different average ages.

Bearing the caveat in mind that age could be a confounding factor in the analyses, we found that RTL was unrelated to haemoparasite infection, infection intensity or plumage morph. Somewhat surprisingly, we found that body condition index was negatively associated with RTL, with individuals with a lower mass than expected given their tarsus length and sex having a higher RTL. Telomere length has been suggested to be directly or indirectly associated with individual quality, which includes body size and foraging behaviour, with longer telomeres typically associated with larger individuals [79–81], which is clearly not the case in our black sparrowhawk cohort. It is possible that larger individuals may start breeding earlier and may breed more frequently, which could account for the shorter telomeres observed in larger individuals, as an increased reproductive effort is associated with increased telomere attrition in several bird species [33,82–84]. However, the interpretation of these results is complicated by the fact that telomere length and condition index in this study were measured only once at a discrete point in time, and it is thus unclear if they reflect long-term telomere dynamics or body condition over time. Furthermore, it is still unclear whether telomere length constitutes a reliable proxy for individual quality in this species, and further research will be required in this regard.

Black sparrowhawks are distributed clinally in South Africa, with more dark morphs in the southwest where breeding occurs during the wet winter season [85,86]. It has been hypothesized that morph distribution may be an adaptive response to variation in parasite prevalence driven by rainfall during the breeding season, assuming a fitness cost of high parasite load during this energetically demanding time [54]. However, while we observed the same pattern of higher haemoparasite infection intensity in light versus dark morph birds as previously reported [53,54], there was no significant correlation between RTL and either infection intensity or plumage morph. These data suggest that in adult black sparrowhawks, any physiological cost of haemoparasite infection does not include telomere shortening and any associated negative traits. It is therefore uncertain whether a lower parasite infection intensity in dark morph birds could have a large enough impact to constitute a selective advantage. This is in line with recent work by McCarren *et al*. [54], which showed that haemoparasite infection intensity was not associated with breeding performance or survival, nor was it higher in the southwest than elsewhere in South Africa. Collectively, these findings cast doubt on the adaptive significance of the lower haemoparasite infection intensities observed in dark morph birds and suggest that this does not explain the higher occurrence of dark morphs in this region.

We found that males had a longer RTL than females. In birds, males are the homogametic sex, and in common with many other raptors, female black sparrowhawks are considerably larger than male birds, with males weighing around 40% less than females [87]. This size difference is particularly pronounced in accipiter hawks [88]. The longer RTL in male black sparrowhawks is thus in line with the predictions of both the heterogametic disadvantage hypothesis, which posits that telomeric maintenance alleles are carried on the homogametic sex chromosome [78,89], and the size selection hypothesis, which posits that the larger-bodied sex will have shorter telomeres due to increased cell division [90]. A recent meta-analysis has suggested that, in vertebrates, the homogametic sex lives longer than the heterogametic sex, though this effect was smaller when females were the heterogametic sex, as in birds [91]. However, this ‘lifespan dimorphism’ does not appear to be related to sex-specific differences in telomere length. An analysis of 51 vertebrates revealed no evidence of consistent differences in the telomere length between adult males and females in any group. In birds, telomere length was significantly longer in ZZ males versus ZW females in just 4 out of the 28 species analysed, and no significant effect of sexual size dimorphism on telomere length was apparent either [92].

Several studies have suggested that plumage polymorphism may be associated with differences in telomere length [39–41]. However, in the black sparrowhawk, we did not find any significant difference in RTL between light and dark morph birds. This suggests that any pleiotropic traits associated with morph in this species do not affect telomere length, and thus negative effects potentially associated with increased telomere attrition such as premature death, lower reproductive success or survival rates and poorer quality offspring would not be expected to be associated with plumage morph in the black sparrowhawk.

A limitation of our study is that the haemoparasite infection and RTL, for most of our sampled birds, were measured at a single point in time, and thus it is not possible to capture any temporal dynamics in either variable. In a related species, the European sparrowhawk (*Accipiter nisus*), haemoparasite infection was found to commence at early life and largely persist throughout an individual’s life (although no clear cases of haemoparasite-induced mortality were seen in the population) [74], but the dynamics of haemoparasite infection are poorly understood in the black sparrowhawk, including how long it takes individuals to control high-intensity infections. For example, it is possible that an individual with a low chronic haemoparasite infection intensity and low RTL at the time of sampling might have recovered from a previous severe infection which resulted in telomere attrition, although it is unclear whether high haemoparasite infection intensity would last for a long enough time to have a measurable effect on the telomere length. While such dynamics would obscure the hypothetical connection between haemoparasite infection intensity and telomere attrition, it should be noted that blood parasite prevalence does not change throughout an individual’s life once an infection has occurred, as opposed to infection intensity which varies over time [93]. Furthermore, note that McCarren *et al.* [54] expressed similar limitations in their study of productivity and survival in relation to haemoparasite infection, as individuals could also only be sampled once. While longitudinal studies would be valuable in addressing this limitation, they would be extremely challenging in a wild raptor population, where the probability of capturing an adult bird drops significantly following an initial capture and handling experience.

## 5. Conclusion

In this study, we confirmed using a qPCR-based methodology that plumage polymorphism in the black sparrowhawk is associated with a possible pleiotropic trait involving immunity, with dark morphs experiencing a significantly lower infection intensity when infected by haemoparasites than light morphs. This may reflect a stronger or more active immune response to infection, possibly associated with pleiotropic effects of the melanocortin system due to the relatively higher production of eumelanin in dark morph birds. However, while this study confirmed the morph-specific difference in infection intensity reported by Lei *et al.* [53], it revealed no association between RTL and either haemoparasite prevalence or intensity, mirroring the results on productivity and survival of McCarren *et al*. [54]. As such, the physiological effects of haemoparasite infection remain unclear, as does the question of whether the dark morph of this species derives any selective advantage from lower haemoparasite infection intensity. Furthermore, while we found that males had a significantly longer RTL than females, no difference was observed between morphs, implying that there is no pleiotropic effect of plumage morph on telomere length. In conclusion, although a pleiotropic effect of morph on immune function in the species continues to be supported, the mechanism by which immunity and melanin pigmentation are linked remains unclear.

## Data Availability

All experimental data generated during this project and the R code used to analyse it can be accessed at [64]. Supplementary data are available online [94].

## References

[1] Paaby AB , Rockman MV . 2013 The many faces of pleiotropy. Trends Genet. **29** , 66–73. (10.1016/j.tig.2012.10.010) 23140989 PMC3558540

[2] Côte J , Boniface A , Blanchet S , Hendry AP , Gasparini J , Jacquin L . 2018 Melanin-based coloration and host-parasite interactions under global change. Proc. R. Soc. B Biol. Sci. **285** , 20180285. (10.1098/rspb.2018.0285) PMC5998088 29848644

[3] Ducrest AL , Keller L , Roulin A . 2008 Pleiotropy in the melanocortin system, coloration and behavioural syndromes. Trends Ecol. Evol. **23** , 502–510. (10.1016/j.tree.2008.06.001) 18644658

[4] Gasparini J , Bize P , Piault R , Wakamatsu K , Blount JD , Ducrest AL , Roulin A . 2009 Strength and cost of an induced immune response are associated with a heritable melanin-based colour trait in female tawny owls. J. Anim. Ecol. **78** , 608–616. (10.1111/j.1365-2656.2008.01521.x) 19175442

[5] Roulin A . 2004 The evolution, maintenance and adaptive function of genetic colour polymorphism in birds. Biol. Rev. Camb. Philos. Soc. **79** , 815–848. (10.1017/s1464793104006487) 15682872

[6] Roulin A , Jungi TW , Pfister H , Dijkstra C . 2000 Female barn owls (Tyto alba) advertise good genes. Proc. R. Soc. Lond. B **267** , 937–941. (10.1098/rspb.2000.1093) PMC1690619 10853738

[7] Jacquin L , Lenouvel P , Haussy C , Ducatez S , Gasparini J . 2011 Melanin‐based coloration is related to parasite intensity and cellular immune response in an urban free living bird: the feral pigeon Columba livia. J. Avian Biol. **42** , 11–15. (10.1111/j.1600-048X.2010.05120.x)

[8] Gangoso L , Grande JM , Ducrest AL , Figuerola J , Bortolotti GR , Andrés JA , Roulin A . 2011 MC1R-dependent, melanin-based colour polymorphism is associated with cell-mediated response in the Eleonora’s falcon. J. Evol. Biol. **24** , 2055–2063. (10.1111/j.1420-9101.2011.02336.x) 21696477

[9] Gangoso L , Gutiérrez-López R , Martínez-de la Puente J , Figuerola J . 2016 Genetic colour polymorphism is associated with avian malarial infections. Biol. Lett. **12** , 20160839. (10.1098/rsbl.2016.0839) 28003524 PMC5206593

[10] Galeotti P , Sacchi R . 2003 Differential parasitaemia in the tawny owl (Strix aluco): effects of colour morph and habitat. J. Zool. **261** , 91–99. (10.1017/S0952836903003960)

[11] Blackburn EH . 1991 Structure and function of telomeres. Nature **350** , 569–573. (10.1038/350569a0) 1708110

[12] Zakian VA . 1995 Telomeres: beginning to understand the end. Science **270** , 1601–1607. (10.1126/science.270.5242.1601) 7502069

[13] Olovnikov AM . 1996 Telomeres, telomerase, and aging: origin of the theory. Exp. Gerontol. **31** , 443–448. (10.1016/0531-5565(96)00005-8) 9415101

[14] Prowse KR , Greider CW . 1995 Developmental and tissue-specific regulation of mouse telomerase and telomere length. Proc. Natl Acad. Sci. USA **92** , 4818–4822. (10.1073/pnas.92.11.4818) 7761406 PMC41798

[15] Meyne J , Ratliff RL , Moyzis RK . 1989 Conservation of the human telomere sequence (TTAGGG)n among vertebrates. Proc. Natl Acad. Sci. USA **86** , 7049–7053. (10.1073/pnas.86.18.7049) 2780561 PMC297991

[16] Hande MP , Samper E , Lansdorp P , Blasco MA . 1999 Telomere length dynamics and chromosomal instability in cells derived from telomerase null mice. J. Cell Biol. **144** , 589–601. (10.1083/jcb.144.4.589) 10037783 PMC2132934

[17] Smogorzewska A , de Lange T . 2002 Different telomere damage signaling pathways in human and mouse cells. EMBO J. **21** , 4338–4348. (10.1093/emboj/cdf433) 12169636 PMC126171

[18] Kirk KE , Harmon BP , Reichardt IK , Sedat JW , Blackburn EH . 1997 Block in anaphase chromosome separation caused by a telomerase template mutation. Science **275** , 1478–1481. (10.1126/science.275.5305.1478) 9045613

[19] Wellinger RJ . 2014 In the end, what’s the problem? Mol. Cell. **53** , 855–856. (10.1016/j.molcel.2014.03.008) 24656125

[20] Haussmann MF , Vleck CM , Nisbet ICT . 2003 Calibrating the telomere clock in common terns, Sterna hirundo. Exp. Gerontol. **38** , 787–789. (10.1016/s0531-5565(03)00109-8) 12855288

[21] Blasco MA . 2005 Telomeres and human disease: ageing, cancer and beyond. Nat. Rev. Genet. **6** , 611–622. (10.1038/nrg1656) 16136653

[22] Bize P , Criscuolo F , Metcalfe NB , Nasir L , Monaghan P . 2009 Telomere dynamics rather than age predict life expectancy in the wild. Proc. R. Soc. B **276** , 1679–1683. (10.1098/rspb.2008.1817) PMC2660992 19324831

[23] Barrett ELB , Burke TA , Hammers M , Komdeur J , Richardson DS . 2013 Telomere length and dynamics predict mortality in a wild longitudinal study. Mol. Ecol. **22** , 249–259. (10.1111/mec.12110) 23167566

[24] Angelier F , Costantini D , Blévin P , Chastel O . 2018 Do glucocorticoids mediate the link between environmental conditions and telomere dynamics in wild vertebrates? A review. Gen. Comp. Endocrinol. **256** , 99–111. (10.1016/j.ygcen.2017.07.007) 28705731

[25] Biard C , Brischoux F , Meillère A , Michaud B , Nivière M , Ruault S , Vaugoyeau M , Angelier F . 2017 Growing in cities: an urban penalty for wild birds? A study of phenotypic differences between urban and rural great tit chicks (Parus major). Front. Ecol. Evol. **5** , 79. (10.3389/fevo.2017.00079)

[26] Herborn KA , Heidinger BJ , Boner W , Noguera JC , Adam A , Daunt F , Monaghan P . 2014 Stress exposure in early post-natal life reduces telomere length: an experimental demonstration in a long-lived seabird. Proc. R. Soc. B **281** , 20133151. (10.1098/rspb.2013.3151) PMC3973262 24648221

[27] Tricola GM et al . 2018 The rate of telomere loss is related to maximum lifespan in birds. Phil. Trans. R. Soc. B **373** 20160445, (10.1098/rstb.2016.0445) 29335369 PMC5784065

[28] Epel ES , Blackburn EH , Lin J , Dhabhar FS , Adler NE , Morrow JD , Cawthon RM . 2004 Accelerated telomere shortening in response to life stress. Proc. Natl Acad. Sci. USA **101** , 17 (10.1073/pnas.0407162101) PMC534658 15574496

[29] von Zglinicki T . 2002 Oxidative stress shortens telomeres. Trends Biochem. Sci. **27** , 339–344. (10.1016/S0968-0004(02)02110-2) 12114022

[30] Salmón P , Nilsson JF , Nord A , Bensch S , Isaksson C . 2016 Urban environment shortens telomere length in nestling great tits, Parus major. Biol. Lett. **12** , 20160155. (10.1098/rsbl.2016.0155) 27303051 PMC4938044

[31] Watson H , Bolton M , Monaghan P . 2015 Variation in early-life telomere dynamics in a long-lived bird: links to environmental conditions and survival. J. Exp. Biol. **218** , 668–674. (10.1242/jeb.104265) 25617465 PMC4376192

[32] Asghar M , Hasselquist D , Hansson B , Zehtindjiev P , Westerdahl H , Bensch S . 2015 Chronic infection. Hidden costs of infection: chronic malaria accelerates telomere degradation and senescence in wild birds. Science **347** , 436–438. (10.1126/science.1261121) 25613889

[33] Reichert S , Stier A , Zahn S , Arrivé M , Bize P , Massemin S , Criscuolo F . 2014 Increased brood size leads to persistent eroded telomeres. Front. Ecol. Evol. **2** , 2. (10.3389/fevo.2014.00009)

[34] Sudyka J , Podmokła E , Drobniak SM , Dubiec A , Arct A , Gustafsson L , Cichoń M . 2019 Sex-specific effects of parasites on telomere dynamics in a short-lived passerine—the blue tit. Sci. Nat. **106** , 6. (10.1007/s00114-019-1601-5) PMC6353807 30701351

[35] Merino S , Moreno J , Sanz JJ , Arriero E . 2000 Are avian blood parasites pathogenic in the wild? A medication experiment in blue tits (Parus caeruleus). Proc. R. Soc. Lond. B **267** , 2507–2510. (10.1098/rspb.2000.1312) PMC1690848 11197126

[36] Marzal A , de Lope F , Navarro C , Møller AP . 2005 Malarial parasites decrease reproductive success: an experimental study in a passerine bird. Oecologia **142** , 541–545. (10.1007/s00442-004-1757-2) 15688214

[37] Sol D , Jovani R , Torres J . 2003 Parasite mediated mortality and host immune response explain age-related differences in blood parasitism in birds. Oecologia **135** , 542–547. (10.1007/s00442-003-1223-6) 16228253

[38] de la Puente JM , Merino S , Tomás G , Moreno J , Morales J , Lobato E , García-Fraile S , Belda EJ . 2010 The blood parasite Haemoproteus reduces survival in a wild bird: a medication experiment. Biol. Lett. **6** , 663–665. (10.1098/rsbl.2010.0046) 20181556 PMC2936130

[39] Karell P , Bensch S , Ahola K , Asghar M . 2017 Pale and dark morphs of tawny owls show different patterns of telomere dynamics in relation to disease status. Proc. R. Soc. B **284** , 20171127. (10.1098/rspb.2017.1127) PMC5543233 28747482

[40] Parolini M et al . 2017 Telomere length is reflected by plumage coloration and predicts seasonal reproductive success in the barn swallow. Mol. Ecol. **26** , 6100–6109. (10.1111/mec.14340) 28851004

[41] Morosinotto C , Bensch S , Karell P . 2021 Telomere length in relation to colour polymorphism across life stages in the tawny owl. J. Avian Biol. **52** , 02564. (10.1111/jav.02564)

[42] Brommer JE , Ahola K , Karstinen T . 2005 The colour of fitness: plumage coloration and lifetime reproductive success in the tawny owl. Proc. R. Soc. B **272** , 935–940. (10.1098/rspb.2005.3052) PMC1564093 16024349

[43] Karell P , Ahola K , Karstinen T , Kolunen H , Siitari H , Brommer JE . 2011 Blood parasites mediate morph-specific maintenance costs in a colour polymorphic wild bird. J. Evol. Biol. **24** , 1783–1792. (10.1111/j.1420-9101.2011.02308.x) 21599778

[44] Galván I , Solano F . 2016 Bird integumentary melanins: biosynthesis, forms, function and evolution. Int. J. Mol. Sci. **17** , 520. (10.3390/ijms17040520) 27070583 PMC4848976

[45] Geiger S , Le Vaillant M , Lebard T , Reichert S , Stier A , LE Maho Y , Criscuolo F . 2012 Catching-up but telomere loss: half-opening the black box of growth and ageing trade-off in wild king penguin chicks. Mol. Ecol. **21** , 1500–1510. (10.1111/j.1365-294X.2011.05331.x) 22117889

[46] Stier A , Massemin S , Zahn S , Tissier ML , Criscuolo F 2015 Starting with a handicap: effects of asynchronous hatching on growth rate, oxidative stress and telomere dynamics in free-living great tits. Oecologia 179, 999–1010. 26314343 10.1007/s00442-015-3429-9

[47] Badás EP , Martínez J , Aguilar Cachafeiro J , Miranda F , Figuerola J , Merino S . 2015 Ageing and reproduction: antioxidant supplementation alleviates telomere loss in wild birds. J. Evol. Biol. **28** , 896–905. (10.1111/jeb.12615) 25758014

[48] Boonekamp JJ , Bauch C , Mulder E , Verhulst S . 2017 Does oxidative stress shorten telomeres? Biol. Lett. **13** , 20170164. (10.1098/rsbl.2017.0164) 28468913 PMC5454244

[49] Reichert S , Stier A . 2017 Does oxidative stress shorten telomeres in vivo? A review. Biol. Lett. **13** , 20170463. 29212750 10.1098/rsbl.2017.0463 PMC5746531

[50] Martin RO , Sebele L , Koeslag A , Curtis O , Abadi F , Amar A . 2014 Phenological shifts assist colonisation of a novel environment in a range‐expanding raptor. Oikos **123** , 1457–1468. (10.1111/oik.01058)

[51] Hockey P , Dean W , Ryan P . 2005 Roberts' birds of Southern Africa, 7th edn. Cape Town, South Africa: The trustees of the John Voelcker bird book fund.

[52] Tate G , Sumasgutner P , Koeslag A , Amar A . 2017 Pair complementarity influences reproductive output in the polymorphic black sparrowhawk Accipiter melanoleucus. J. Avian Biol. **48** , 387–398. (10.1111/jav.01100)

[53] Lei B , Amar A , Koeslag A , Gous TA , Tate GJ . 2013 Differential haemoparasite intensity between black sparrowhawk (Accipiter melanoleucus) morphs suggests an adaptive function for polymorphism. PLoS One **8** , e81607. (10.1371/journal.pone.0081607) 24391707 PMC3876978

[54] McCarren S , Sumasgutner P , Tate G , Koeslag A , Amar A . 2021 Clinal variation in the polymorphic black sparrowhawk Accipiter melanoleucus is unrelated to infection by the blood parasite Haemoproteus nisi. J. Ornithol. **162** , 231–241. (10.1007/s10336-020-01823-3)

[55] Kanai N , Fujii T , Saito K , Tokoyama T . 1994 Rapid and simple method for preparation of genomic DNA from easily obtainable clotted blood. J. Clin. Pathol. **47** , 1043–1044. (10.1136/jcp.47.11.1043) 7829682 PMC503071

[56] Friedl TWP , Groscurth E . 2012 A real-time PCR protocol for simple and fast quantification of blood parasite infections in evolutionary and ecological studies and some data on intensities of blood parasite infections in a subtropical weaverbird. J. Ornithol. **153** , 239–247. (10.1007/s10336-011-0735-9)

[57] Suri J , Sumasgutner P , Hellard É , Koeslag A , Amar A . 2017 Stability in prey abundance may buffer black sparrowhawks Accipiter melanoleucus from health impacts of urbanization. Ibis. **159** , 38–54. (10.1111/ibi.12422)

[58] Tan TMC , Nelson JS , Ng HC , Ting RCY , Kara UAK . 1997 Direct PCR amplification and sequence analysis of extrachromosomal plasmodium DNA from dried blood spots. Acta Tropica. **68** , 105–114. (10.1016/S0001-706X(97)00080-6) 9352006

[59] Criscuolo F , Bize P , Nasir L , Metcalfe NB , Foote CG , Griffiths K , Gault EA , Monaghan P . 2009 Real‐time quantitative PCR assay for measurement of avian telomeres. J. Avian Biol. **40** , 342–347. (10.1111/j.1600-048X.2008.04623.x)

[60] Bejerano G , Pheasant M , Makunin I , Stephen S , Kent WJ , Mattick JS , Haussler D . 2004 Ultraconserved elements in the human genome. Science **304** , 1321–1325. (10.1126/science.1098119) 15131266

[61] Asghar M , Hasselquist D , Bensch S . 2011 Are chronic avian haemosporidian infections costly in wild birds? J. Avian Biol. **42** , 530–537. (10.1111/j.1600-048X.2011.05281.x)

[62] Arriero E , Pérez-Tris J , Ramírez A , Remacha C . 2018 Trade-off between tolerance and resistance to infections: an experimental approach with malaria parasites in a passerine bird. Oecologia **188** , 1001–1010. (10.1007/s00442-018-4290-4) 30377770

[63] Asghar M , Westerdahl H , Zehtindjiev P , Ilieva M , Hasselquist D , Bensch S . 2012 Primary peak and chronic malaria infection levels are correlated in experimentally infected great reed warblers. Parasitology **139** , 1246–1252. (10.1017/S0031182012000510) 22716664

[64] Ingle R , *et al* . 2024 Data from: Pleiotropic effects of melanin pigmentation: haemoparasite infection intensity but not telomere length is associated with morph in black sparrowhawks. Dryad Digital Repository. (10.5061/dryad.vhhmgqp21) PMC10987988 38577209

[65] Jakob EM , Marshall SD , Uetz GW . 1996 Estimating fitness: a comparison of body condition indices. Oikos **77** , 61. (10.2307/3545585)

[66] Krebs CJ , Singleton GR . 1993 Indexes of condition for small mammals. Aust. J. Zool. **41** , 317–323. (10.1071/ZO9930317)

[67] Reist JD . 1985 An empirical evaluation of several univariate methods that adjust for size variation in morphometric data. Can. J. Zool. **63** , 1429–1439. (10.1139/z85-213)

[68] Bartoń K . 2023 MuMIn: Multi-Model Inference. See https://cran.hafro.is/web/packages/MuMIn/MuMIn.pdf

[69] Akaike H . 1998 Information theory and an extension of the maximum likelihood principle. In Selected papers of Hirotugu Akaike pp. 199–213. New York, NY: Springer.

[70] Burnham KP , Anderson DR , Burnham KP , Anderson DR . 1998 Practical use of the information-theoretic approach. New York, NY: Springer. (10.1007/978-1-4757-2917-7)

[71] Verhulst S , Aviv A , Benetos A , Berenson GS , Kark JD . 2013 Do leukocyte telomere length dynamics depend on baseline telomere length? An analysis that corrects for 'regression to the mean'. Eur. J. Epidemiol. **28** , 859–866. (10.1007/s10654-013-9845-4) 23990212

[72] R Core Team . 2019 R: A language and environment for statistical computing. Vienna, Austria: R Foundation for Statistical Computing. See https://www.R-project.org/

[73] Bates D , Mächler M , Bolker B , Walker S. 2015 Fitting linear mixed-effects models using lme4. J. Stat. Softw. 67, 1–48. (10.18637/jss.v067.i01)

[74] Svobodová M *et al* . 2023 Blood parasites (Trypanosoma, Leucocytozoon, Haemoproteus) in the Eurasian sparrowhawk (Accipiter nisus): diversity, incidence and persistence of infection at the individual level. Parasit. Vectors. **16** , 15. (10.1186/s13071-022-05623-x) 36641440 PMC9840293

[75] Salomons HM , Mulder GA , van de Zande L , Haussmann MF , Linskens MHK , Verhulst S . 2009 Telomere shortening and survival in free-living corvids. Proc. R. Soc. B **276** , 3157–3165. (10.1098/rspb.2009.0517) PMC2817122 19520803

[76] Hall ME , Nasir L , Daunt F , Gault EA , Croxall JP , Wanless S , Monaghan P . 2004 Telomere loss in relation to age and early environment in long-lived birds. Proc. R. Soc. Lond. B **271** , 1571–1576. (10.1098/rspb.2004.2768) PMC1691772 15306302

[77] Horn T , Robertson BC , Gemmell NJ . 2010 The use of telomere length in ecology and evolutionary biology. Heredity (Edinb). **105** , 497–506. (10.1038/hdy.2010.113) 20736972

[78] Barrett ELB , Richardson DS . 2011 Sex differences in telomeres and lifespan. Aging Cell. **10** , 913–921. (10.1111/j.1474-9726.2011.00741.x) 21902801

[79] Le Vaillant M , Viblanc VA , Saraux C , Le Bohec C , Le Maho Y , Kato A , Criscuolo F , Ropert-Coudert Y . 2015 Telomere length reflects individual quality in free-living adult king penguins. Polar Biol. **38** , 2059–2067. (10.1007/s00300-015-1766-0)

[80] Bauch C , Becker PH , Verhulst S . 2013 Telomere length reflects phenotypic quality and costs of reproduction in a long-lived seabird. Proc. R. Soc. B **280** , 20122540. (10.1098/rspb.2012.2540) PMC3574312 23222450

[81] Angelier F , Weimerskirch H , Barbraud C , Chastel O . 2019 Is telomere length a molecular marker of individual quality? Insights from a long‐lived bird. Funct. Ecol. **33** , 1076–1087. (10.1111/1365-2435.13307)

[82] Sudyka J . 2019 Does reproduction shorten telomeres? Towards integrating individual quality with life-history strategies in telomere biology. Bioessays **41** , e1900095. (10.1002/bies.201900095) 31577044

[83] Sudyka J , Arct A , Drobniak SM , Gustafsson L , Cichoń M. 2019 Birds with high lifetime reproductive success experience increased telomere loss. Biol. Lett. 15, 20180637. (10.1098/rsbl.2018.0637) 30958221 PMC6371901

[84] Sudyka J , Arct A , Drobniak S , Dubiec A , Gustafsson L , Cichoń M. 2014 Experimentally increased reproductive effort alters telomere length in the blue tit (Cyanistes caeruleus). J. Evol. Biol. 27, 2258–2264. (10.1111/jeb.12479) 25228433

[85] Amar A , Koeslag A , Malan G , Brown M , Wreford E . 2014 Clinal variation in the morph ratio of black sparrowhawks Accipiter melanoleucus in South Africa and its correlation with environmental variables. Ibis (Lond. 1859). **156** , 627–638. (10.1111/ibi.12157)

[86] Tate GJ , Bishop JM , Amar A. 2016 Differential foraging success across a light level spectrum explains the maintenance and spatial structure of colour morphs in a polymorphic bird. Ecol. Lett. 19, 679–686. (10.1111/ele.12606) 27132885

[87] Amar A , Koeslag A , Curtis O . 2013 Plumage polymorphism in a newly colonized black sparrowhawk population: classification, temporal stability and inheritance patterns. J. Zool. **289** , 60–67. (10.1111/j.1469-7998.2012.00963.x)

[88] Ferguson-Lees J , Christie DA , Franklin K , Mead D , Burton P . 2001 Raptors of the world. London, UK: Christopher Helm.

[89] Marais GAB , Gaillard JM , Vieira C , Plotton I , Sanlaville D , Gueyffier F , Lemaitre JF . 2018 Sex gap in aging and longevity: can sex chromosomes play a role? Biol. Sex Differ. **9** , 1–14. (10.1186/s13293-018-0181-y) 30016998 PMC6050741

[90] Stindl R . 2004 Tying it all together: telomeres, sexual size dimorphism and the gender gap in life expectancy. Med. Hypotheses. **62** , 151–154. (10.1016/s0306-9877(03)00316-5) 14729022

[91] Xirocostas ZA , Everingham SE , Moles AT . 2020 The sex with the reduced sex chromosome dies earlier: a comparison across the tree of life. Biol. Lett. **16** , 20190867. (10.1098/rsbl.2019.0867) 32126186 PMC7115182

[92] Remot F , Ronget V , Froy H , Rey B , Gaillard JM , Nussey DH , Lemaître JF . 2020 No sex differences in adult telomere length across vertebrates: a meta-analysis. R. Soc. Open Sci. **7** , 200548. (10.1098/rsos.200548) 33391781 PMC7735339

[93] Valkiunas G . 2004 Avian malaria parasites and other Haemosporidia. Boca Raton, FL: CRC press. (10.1201/9780203643792)

[94] Rodseth E , Sumasgutner P , Tate G , Nilsson JF , Watson H , Maritz M , et al . 2024 Data from: Pleiotropic effects of melanin pigmentation: haemoparasite infection intensity but not telomere length is associated with morph in black sparrowhawks. Figshare. (10.6084/m9.figshare.c.7095871) PMC10987988 38577209

